# Statistical Feature Engineering for Robot Failure Detection: A Comparative Study of Machine Learning and Deep Learning Classifiers

**DOI:** 10.3390/s26051649

**Published:** 2026-03-05

**Authors:** Sertaç Savaş

**Affiliations:** Department of Mechatronics Engineering, Erciyes University, 38039 Kayseri, Türkiye; sertacsavas@erciyes.edu.tr

**Keywords:** robot failure detection, classification, feature engineering, statistical features, machine learning, deep learning

## Abstract

Industrial robots are widely used in critical tasks such as assembly, welding, and material handling as core components of modern manufacturing systems. For the reliable operation of these systems, early and accurate detection of execution failures is crucial. In this study, a comprehensive comparison of machine learning and deep learning methods is conducted for the classification of robot execution failures using data acquired from force–torque sensors. Three different feature engineering approaches are proposed. The first is a Baseline approach that includes 90 raw time-series features. The second is the Domain-6 approach, which consists of 6 basic statistical features per sensor (36 in total). The third is the Domain-12 approach, which comprises 12 comprehensive statistical features per sensor (72 in total). The domain features include the mean, standard deviation, minimum, maximum, range, slope, median, skewness, kurtosis, RMS, energy, and IQR. In total, ten classification algorithms are evaluated, including eight machine learning methods and two deep learning models: Support Vector Machines (SVM), Random Forest (RF), k-Nearest Neighbors (KNN), Artificial Neural Network (ANN), Naive Bayes (NB), Decision Trees (DT), eXtreme Gradient Boosting (XGBoost), Light Gradient Boosting Machine (LightGBM-LGBM), as well as a One-Dimensional Convolutional Neural Network (CNN-1D) and Long Short-Term Memory (LSTM). For traditional machine learning algorithms, 5 × 5 nested cross-validation is used, whereas for deep learning models, 5-fold cross-validation with a 20% validation split is employed. To ensure statistical reliability, all experiments are repeated over 30 independent runs. The experimental results demonstrate that feature engineering has a decisive impact on classification performance. In addition, regardless of the feature set, the highest accuracy (93.85% ± 0.90) is achieved by the Naive Bayes classifier using the Baseline features. The Domain-12 feature set provides consistent improvements across many algorithms, with substantial performance gains. The results are reported using accuracy, precision, recall, and F1-score metrics and are supported by confusion matrices. Finally, permutation feature importance analysis indicates that the skewness features of the Fx and Fy sensors are the most critical variables for failure detection. Overall, these findings show that time-domain statistical features offer an effective approach for robot failure classification.

## 1. Introduction

Industrial robots have become indispensable components of modern manufacturing systems. The reliability of these systems—widely deployed in applications such as assembly, welding, painting, and material handling—is critical for production efficiency and occupational safety. Failures in robotic systems can lead to production downtime, quality defects, and potential safety hazards. Therefore, early and accurate detection of robot failures is a fundamental requirement for the effective management of industrial automation systems.

Force–torque sensors play an important role in detecting failures in robot execution. By measuring interactions between the robot arm and its environment, these sensors enable differentiation between normal operation and abnormal conditions. However, extracting meaningful information from sensor signals and performing failure classification requires appropriate feature engineering and careful selection of classification algorithms. In conventional approaches, raw sensor data are used directly, which often results in high dimensionality and increased computational complexity.

In the study using the robot execution failure dataset introduced by Seabra Lopes and Camarinha-Matos [1], various feature transformation strategies were evaluated for robot failure classification. Windowed averages, derivatives, and monotonicity measures were used, yielding substantial improvements in accuracy. However, that study was conducted with a limited set of algorithms, such as SKIL and OC1, and it did not assess modern machine learning techniques. Today, powerful classifiers—including support vector machines, random forests, gradient boosting methods, and deep learning algorithms—are widely used in fault detection problems. A comprehensive evaluation of the effectiveness of these modern approaches for robot failure classification would therefore represent a meaningful contribution to the field. In addition, performing classification using statistical features spanning both basic and advanced descriptors offers an important perspective on feature engineering.

The literature on fault detection and predictive maintenance in industrial robots has evolved within a broad framework that emphasizes approaches focusing on robot subcomponents (e.g., motor drives and joints), deep learning-based feature extraction from sensor data, and Internet of Robotic Things (IoRT)-based online monitoring architectures. Eang and Lee [2] developed a CNN-RNN (Recurrent Neural Network) hybrid observer framework for predictive maintenance and fault detection in DC motor drives of industrial robots, aiming to learn dynamic characteristics from sensor data to support early fault identification and maintenance. Bilal et al. [3] proposed an IoRT-based monitoring architecture for online fault diagnosis in industrial robot joints; their approach employed a Transfer Learning (TL)-assisted deep learning model to identify joint fault conditions across varying operating regimes reliably. Liu et al. [4] introduced a Dilated-CNN-based method for cross-axis fault diagnosis in multi-axis industrial robots, strengthening the model with Self-Attention and using TL to adapt it to different axis data to mitigate limited-data challenges. Pan et al. [5] developed a data-driven Deep Convolutional Neural Network (DCNN) approach for diagnosing sensor and actuator faults in robot joints, aiming to classify different fault types by learning discriminative representations from fused sensor–actuator data. Sabry et al. [6] proposed a fault detection approach for industrial robots based on comparing power consumption and encoder data between a “healthy reference” and real-time measurements, aiming to distinguish joint/encoder degradations by representing power-consumption patterns with a simple modeling scheme. Shi et al. [7] developed an Interacting Multiple Model-based Adaptive Control System for stable steering of distributed-drive electric vehicles under various road excitations, aiming to reduce instabilities (e.g., oversteer/understeer) by adapting the vehicle model to conditions while preserving yaw and longitudinal stability. Li et al. [8] proposed a deep learning approach supported by Data Augmentation (DA) for fault diagnosis in rotating machinery, aiming to improve diagnostic performance with limited labeled data by generating richer training samples via simple signal-level augmentation operations.

Similarly, the fault diagnosis literature for industrial power transmission elements, such as rotating machinery and bearings, has focused on addressing limited-labeled-data issues through data augmentation, leveraging hybrid CNN–LSTM architectures to exploit time and frequency information jointly, and achieving reliable diagnosis via signal-based methods such as Motor Current Signature Analysis (MCSA). Fu et al. [9] developed a parallel CNN–LSTM deep learning model for bearing fault diagnosis from vibration signals, aiming to achieve more robust feature extraction and reliable diagnosis by jointly leveraging time and frequency information. Chen et al. [10] proposed a Multi-Scale CNN–LSTM model to automatically learn features directly from raw vibration data for bearing fault diagnosis. Han et al. [11] aimed to jointly learn spatiotemporal features by combining CNN, LSTM, and Gated Recurrent Unit (GRU) architectures for bearing fault diagnosis. Rohan et al. [12] investigated the diagnosis of Rotate Vector (RV) reducer faults in industrial robots using motor current signals and MCSA. Raouf et al. [13] proposed an MCSA-based data-driven classification approach for identifying robotic RV reducer faults from motor current. Fu et al. [14] presented a lightweight, edge-deployable real-time fault-diagnosis method for power transformers based on acoustic signals. Li et al. [15] proposed an MCSA-based approach for local fault diagnosis in robotic rotary vector reducers.

In this study, a comprehensive classification investigation is conducted for detecting robot execution failures. The main contributions can be summarized as follows: (1) Inspired by the modern signal processing literature, a new time-domain feature set is proposed, including statistical descriptors such as mean, standard deviation (std), minimum, maximum, range, slope, median, skewness, kurtosis, root mean square (RMS), energy, and interquartile range (IQR). (2) A total of ten classifiers are comprehensively compared, including eight conventional machine learning methods (SVM, RF, KNN, ANN, NB, DT, XGBoost, LGBM) and two deep learning models (CNN-1D, LSTM). (3) For reliable performance evaluation, 5 × 5 nested cross-validation and 30 independent runs are used for conventional machine learning models. In contrast, for deep learning models, 5-fold outer cross-validation with early stopping is employed, again with 30 independent runs. (4) Feature-related outcomes are analyzed within the Permutation Feature Importance framework.

## 2. Materials and Methods

**Figure 1:**
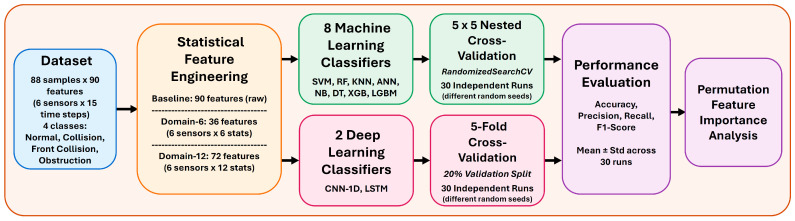
Flowchart of the methodology.

This section presents the study’s methodological framework in detail. First, the dataset is introduced. In the domain-based feature extraction subsection, three feature sets (Baseline, Domain-6, and Domain-12) are described, and the mathematical formulations of the 12 statistical features are provided. Next, the theoretical foundations and operating principles of the ten classification algorithms are summarized. Within the cross-validation strategy, nested cross-validation and k-fold cross-validation approaches are explained. Finally, the hyperparameter search spaces and the most frequently selected parameter values are reported, and the evaluation metrics used for performance assessment—accuracy, precision, recall, and F1-score—are defined. The methodological flow diagram of the study is shown in Figure 1.

### 2.1. Dataset Description and Preprocessing

In this study, the “Robot Execution Failures” dataset available in the UCI Machine Learning Repository is used [16]. The dataset was obtained from experiments conducted on an industrial robot arm, where force–torque sensor data were collected during a pick-and-place macro-operation. In this work, the LP1 (Approach to Grasp) subset is considered. This subset contains failures occurring while the robot approaches a part to grasp it, and its main characteristics are summarized in Table 1.

The matrix in Equation (1) represents the input sample structure of the dataset.

(1) $$\left[\begin{array}{cccccc} Fx1 & Fy1 & Fz1 & Tx1 & Ty1 & Tz1\\ Fx2 & Fy2 & Fz2 & Tx2 & Ty2 & Tz2\\ \vdots & \vdots & \vdots & \vdots & \vdots & \vdots \\ Fx15 & Fy15 & Fz15 & Tx15 & Ty15 & Tz15 \end{array}\right]$$

where Fx1 to Fx15 denote the temporal evolution of the force component Fx within the observation window; the same notation applies to Fy, Fz, and the torque components. Overall, the raw input contains 90 features (6 sensor channels × 15 time steps). The class distribution of the dataset is reported in Table 2. The dataset includes normal operation and three different failure types.

Z-score standardization is applied for feature normalization, transforming each feature to have zero mean and unit variance. To prevent data leakage, normalization is performed separately for each fold within the cross-validation loop: the StandardScaler parameters (mean and standard deviation) are computed exclusively from the training data, and then the transform is applied to both training and test sets. This approach ensures that test data do not contribute to the calculation of scaling parameters, thereby preserving cross-validation integrity. The normalization formula is given in Equation (2).

(2) $$z=\frac{x-\mu }{\sigma }$$

Here, x denotes the raw feature value, μ is the feature mean, σ is the standard deviation, and z represents the normalized value.

### 2.2. Domain-Based Feature Extraction

In this study, the term domain features refers to handcrafted time-domain statistical descriptors extracted from each force–torque sensor channel, rather than raw time-series samples. The Baseline set uses raw measurements directly, whereas the Domain-6 and Domain-12 sets are obtained by computing statistical features per sensor channel. Domain-6 includes first-order statistics that are simple to compute and interpret (mean, standard deviation, minimum, maximum, range, and slope), while Domain-12 extends these with distribution-shape measures (skewness and kurtosis), robust statistics (median and IQR), and signal-energy measures (RMS and energy). In the context of robot execution failure detection, these statistics can be considered domain-informed because they capture physically meaningful characteristics of force–torque signals and provide a more compact and interpretable representation than raw samples. The feature sets and their dimensions are summarized in Table 3, and the complete list of statistical features is provided in Table 4.

The mean feature represents the central tendency of the time series and is computed using the arithmetic mean definition given in Equation (3).

(3) $$\mu =\frac{1}{n}\sum_{i=1}^{n}x_{i}$$

where $x_{i}$ is the i-th sample value, n is the number of samples, and μ denotes the mean. The standard deviation, given in Equation (4), measures the dispersion of the data around the mean.

(4) $$\sigma =\sqrt{\frac{1}{n}\sum_{i=1}^{n}{(x_{i}-\mu )}^{2}}$$

where σ is the standard deviation, μ is the mean, $x_{i}$ is the i-th sample, and n is the number of samples. The minimum feature in Equation (5) denotes the smallest observation in the sequence, the maximum feature in Equation (6) denotes the largest observation, and the range feature in Equation (7) is defined as the difference between the maximum and minimum values.

(5) $$x_{min}=\min\left(x_{1},\ldots ,x_{n}\right)$$

(6) $$x_{max}=\max\left(x_{1},\ldots ,x_{n}\right)$$

(7) $$R=x_{max}-x_{min}$$

where $x_{min}$ is the minimum observation, $x_{max}$ is the maximum observation, and R is the range value (i.e., the max–min difference). The slope feature is computed by fitting a first-order polynomial (linear fit) to the time-series data using the least-squares method. The slope coefficient is obtained via the linear regression slope expression in Equation (8).

(8) $$m=\frac{\sum_{i=1}^{n}\left(t_{i}-\bar{t}\right)\left(x_{i}-\bar{x}\right)}{{\sum_{i=1}^{n}\left(t_{i}-\bar{t}\right)}^{2}}$$

where m is the slope coefficient, $t_{i}$ is the i-th time sample, $x_{i}$ is the i-th measurement, $\bar{t}$ is the mean of the time indices, and $\bar{x}$ is the mean of the measurements. The median is the middle value of the ordered data. For odd/even sample sizes, the median is computed using the piecewise definition given in Equation (9).

(9) $$\tilde{x}=\left\{\begin{array}{cc} x_{\left(\frac{n+1}{2}\right)}, & n\;is\;odd\\ \frac{x_{\left(\frac{n}{2}\right)}+x_{\left(\frac{n}{2}+1\right)}}{2}, & n\;is\;even \end{array}\right.$$

where $x_{\left(j\right)}$ denotes the j-th element of the sorted data, n is the number of samples, and $\tilde{x}$ is the median. The k-th central moment is a fundamental quantity used to compute shape descriptors such as skewness and kurtosis, and it is defined as in Equation (10).

(10) $$m_{k}=\frac{1}{n}\sum_{i=1}^{n}{\left(x_{i}-\mu \right)}^{k}$$

where $m_{k}$ is the k-th central moment, k is the moment order, $x_{i}$ is the i-th sample, and μ is the mean. Skewness measures the asymmetry of the distribution and is obtained by normalizing the third central moment with the second central moment, as given in Equation (11).

(11) $$\gamma_{1}=\frac{m_{3}}{m_{2}^{3/2}}$$

where $\gamma_{1}$ is the skewness coefficient, $m_{2}$ is the second central moment, and $m_{3}$ is the third central moment. Kurtosis measures the tail heaviness of the distribution. The excess kurtosis is computed by normalizing the fourth central moment with the second central moment and subtracting 3, as given in Equation (12).

(12) $$\gamma_{2}=\frac{m_{4}}{m_{2}^{2}}-3$$

where $\gamma_{2}$ denotes the excess kurtosis, $m_{4}$ is the fourth central moment, and $m_{2}$ is the second central moment. The RMS represents the effective magnitude of the signal and is computed using the root-mean-square definition in Equation (13).

(13) $$RMS=\sqrt{\frac{1}{n}\sum_{i=1}^{n}x_{i}^{2}}$$

where $x_{i}$ is the i-th sample and n is the number of samples. The energy feature is defined as the sum of squared sample values and is computed as in Equation (14).

(14) $$E=\sum_{i=1}^{n}x_{i}^{2}$$

where E is the total energy and $x_{i}$ is the i-th sample. Finally, the IQR robustly measures the central spread of the distribution and is computed as the difference between the third and first quartiles. This is given in Equation (15).

(15) $$IQR=Q_{3}-Q_{1}$$

where $Q_{1}$ is the first quartile (25th percentile) and $Q_{3}$ is the third quartile (75th percentile).

### 2.3. Classification Algorithms

In this study, ten classification algorithms representing different learning paradigms are evaluated. The algorithms are considered under two main categories: conventional machine learning and deep learning.

SVM is a kernel-based classifier that aims to find the optimal hyperplane maximizing the separation margin between classes. For non-linearly separable problems, kernel functions map the data into a higher-dimensional space to enable linear separation. During hyperparameter optimization, radial basis function (RBF), linear, and polynomial kernels are examined, and the RBF kernel is selected most frequently. Owing to its ability to capture local patterns, the RBF kernel provided the most suitable performance for this dataset. It measures similarity between two samples via an exponential transformation of the Euclidean distance, while the gamma (γ) parameter controls the flexibility of the decision boundary [17].

RF is an ensemble learning method that combines multiple decision trees using bagging (bootstrap aggregating). Each tree is trained independently using a random subset of the training data and a random subset of features. This randomness reduces variance and mitigates overfitting. The final classification is obtained by majority voting across trees; i.e., the class predicted by the largest number of trees is assigned as the output [18].

KNN is an instance-based learning algorithm that does not require explicit model training. For a test sample, the k closest training samples are identified, and the majority class among these neighbors is assigned as the prediction. Performance depends on the choice of k and the distance metric; small k values increase sensitivity to noise, whereas large k values may blur class boundaries [19].

ANNs are feedforward networks inspired by biological neural systems and consist of one or more hidden layers. In this work, a Multi-Layer Perceptron (MLP) architecture is used. Each neuron computes a weighted sum of its inputs and applies a nonlinear activation function. The Rectified Linear Unit (ReLU) activation improves computational efficiency and gradient propagation by setting negative values to zero. Network weights are updated iteratively using backpropagation [20].

NB is a probabilistic classifier based on Bayes’ theorem. It operates under the “naive” assumption that features are conditionally independent given the class label. Although this assumption rarely holds exactly in practice, NB often performs surprisingly well in real-world problems. The Gaussian Naive Bayes variant assumes that continuous features follow a normal distribution within each class and computes posterior probabilities accordingly [21]. In this implementation, prior probabilities are computed from class frequencies in the training set; that is, the prior probability of each class equals the proportion of training samples belonging to that class. Given the class imbalance in the dataset, larger classes have higher prior probabilities, which may influence posterior probability calculations. However, the stratified cross-validation approach employed in this study preserves class proportions in each fold, systematically controlling this effect.

DTs are rule-based classifiers that recursively partition the data according to feature values. Starting from the root node, each split is determined by the most informative feature, and class decisions are made at leaf nodes. In the Classification and Regression Trees (CART) framework, split quality is evaluated using criteria such as Gini impurity or entropy. Gini impurity quantifies the class homogeneity at a node, with lower values indicating purer (more homogeneous) nodes [22].

XGBoost is a high-performance, scalable implementation of gradient boosting. It constructs a strong ensemble by adding weak learners (decision trees) sequentially, where each new tree focuses on correcting errors made by the previous ensemble. XGBoost controls overfitting via regularization terms and accelerates optimization using a second-order Taylor approximation. Practical features such as parallel processing and native handling of missing values contribute to its wide adoption [23].

LGBM is an efficient gradient boosting method optimized for large-scale datasets. It reduces memory usage and speeds up training through histogram-based learning, which discretizes continuous values into bins. Unlike level-wise tree growth, LGBM employs a leaf-wise strategy that splits the leaf that yields the largest reduction in loss. While leaf-wise growth can converge faster, constraints such as maximum depth and number of leaves are important to prevent overfitting [24].

CNN-1D architectures are designed to automatically extract local patterns from time series and signal data. Convolutional layers slide learnable filters (kernels) over the input to detect local features, while pooling layers reduce the dimensionality of the feature map to lower computational cost and introduce translational invariance. In this study, a two-convolution-layer architecture is adopted to capture temporal patterns in the force–torque sensor signals [25].

LSTM networks are specialized recurrent neural networks (RNNs) capable of learning long-term dependencies in sequential data. To address the vanishing gradient problem in conventional RNNs, LSTM introduces a cell state and gating mechanisms (forget, input, and output gates). The forget gate decides which information to discard, the input gate determines what new information to store, and the output gate controls what information is passed to the next time step. This architecture is well-suited for problems where temporal dependencies are critical, such as robot failure detection [26].

### 2.4. Cross-Validation Strategy and Training Settings

In this study, nested cross-validation (NCV) is employed for model evaluation and hyperparameter optimization of the conventional machine learning algorithms (SVM, RF, KNN, ANN, NB, DT, XGBoost, and LGBM) [27]. Unlike standard cross-validation, NCV uses separate loops for hyperparameter selection and performance estimation, thereby providing more reliable generalization estimates. The adopted NCV scheme follows a 5 × 5 configuration. The outer loop uses 5-fold stratified cross-validation to assess generalization performance, where in each outer fold 80% of the data are used for training and 20% for testing. The inner loop is used for hyperparameter optimization by splitting the training set again into 5 folds and selecting the best hyperparameter configuration. Hyperparameter search is conducted using RandomizedSearchCV, with 20 random combinations evaluated from the predefined search space for each algorithm.

To improve statistical reliability, the entire experimental procedure is repeated over 30 independent runs. In each run, the data are shuffled using a different random seed, enabling robust reporting of results that are less sensitive to random variation.

For the deep learning models (CNN-1D and LSTM), a different evaluation strategy is adopted due to the small dataset size (N = 88). Because deep learning hyperparameter tuning on small datasets is prone to overfitting, a fixed-architecture approach is preferred instead of nested cross-validation. On extremely small datasets (N = 88), hyperparameter optimization in nested CV would evaluate configurations on only ~14 validation samples per inner fold, risking overfitting to fold-specific characteristics. The architectures employed are based on established designs from time-series classification literature. Moreover, since this study’s focus is isolating the effect of feature engineering rather than maximizing DL performance, using the same architecture across all feature sets ensures that performance changes reflect feature quality rather than architecture–feature interactions.

As the outer evaluation loop, 5-fold stratified cross-validation is applied, and in each fold, 20% of the training split is reserved for validation. To mitigate overfitting on the small dataset, several strategies are employed: (1) early stopping monitors validation loss and terminates training when no improvement is observed for 5 consecutive epochs, preventing the model from continuing to fit training data after generalization plateaus; (2) 30 independent runs with different random seeds ensure that results are not artifacts of a particular train-test split; (3) stratified 5-fold cross-validation ensures that all samples are used for testing exactly once, providing robust performance estimates. The moderate standard deviations observed in the Results Section across runs indicate consistent generalization rather than memorization. The deep learning training parameters are set to a maximum of 50 epochs and a batch size of 32.

The applied cross-validation strategy and the main training parameters are summarized in Table 5.

### 2.5. Hyperparameter Optimization

For the conventional machine learning algorithms, hyperparameter optimization is performed within the inner loop of nested cross-validation using RandomizedSearchCV. For each algorithm, 20 random configurations are sampled from the predefined search space and evaluated using 5-fold cross-validation. For the deep learning models, due to the small dataset size, a fixed-architecture strategy is adopted, and overfitting is mitigated via early stopping (patience = 5). The hyperparameter ranges and the most frequently selected settings for the machine learning algorithms are presented in Table 6, while the deep learning model architectures are given in Table 7.

### 2.6. Performance Metrics

In this study, model performance is evaluated using four metrics. Accuracy indicates overall success by measuring the proportion of correctly classified samples among all samples. Precision reflects how many of the predicted positive instances are truly positive, thereby capturing the effect of false positives. Recall quantifies the proportion of actual positive instances correctly identified, measuring the model’s ability to detect positive samples. F1-score is the harmonic mean of precision and recall and represents the balance between these two measures; it is particularly important for a more reliable assessment of performance on imbalanced datasets. The metrics and their formulations are provided in Table 8.

Here, TP (True Positive) denotes correctly predicted positives, TN (True Negative) correctly predicted negatives, FP (False Positive) incorrectly predicted positives, and FN (False Negative) incorrectly predicted negatives.

## 3. Results

### 3.1. Classification Results

This section presents the classification results obtained for three feature sets (Baseline, Domain-6, and Domain-12) and ten classification algorithms. For the Baseline feature set, accuracy, precision, recall, and F1-score values, together with the total computation times, are reported in Table 9. Similarly, the corresponding performance metrics and total computation times for the Domain-6 feature set are provided in Table 10, while the results obtained using the Domain-12 feature set are presented in Table 11. In all tables, the metrics are reported as mean ± standard deviation [minimum, maximum], and the reported values represent the outcomes of 30 independent runs for each combination. In Table 9, Table 10 and Table 11, “Total Time” indicates the cumulative computation time (in minutes) for all 30 independent runs of the respective algorithm, including nested cross-validation with RandomizedSearchCV (for ML) or 5-fold CV with early stopping (for DL).

The code is implemented in Python 3.13.5 and executed on a computer running Windows 10 (64-bit) equipped with an Intel Core i7-7700HQ CPU @ 2.80 GHz, 16 GB RAM, and an NVIDIA GTX 1050 (4 GB) GPU and all models are executed using GPU-accelerated implementations.

When the classification results obtained for the three feature sets are examined, it is evident that the performance of the algorithms varies substantially depending on the feature representation. With the Baseline feature set, Naive Bayes achieves the highest accuracy (93.85%), while commonly used algorithms such as RF also produces competitive results. When switching to the Domain-6 feature set, overall improvements are observed for most classifiers, except for Naive Bayes. The Domain-12 feature set, which incorporates advanced statistics such as skewness, kurtosis, energy, and IQR, yields consistent performance gains across many algorithms. In particular, SVM, KNN, and ANN achieve notable improvements with Domain-12. Among the deep learning models, CNN-1D performed relatively poorly with the Baseline feature set; however, with Domain-12, it achieves 90.4% accuracy, second only to Naive Bayes. Overall, the lowest standard deviation values are observed for Naive Bayes and SVM, indicating that these algorithms are more stable across different data splits.

**Figure 2:**
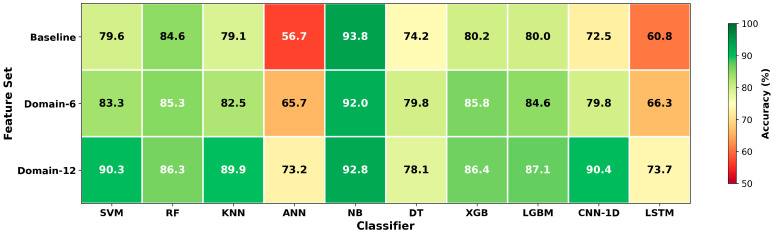
Accuracy heatmap.

Figure 2 presents the accuracy values for the three feature sets and ten classifiers in a heatmap. Dark green tones indicate high accuracy, whereas red tones represent low accuracy.

**Figure 3:**
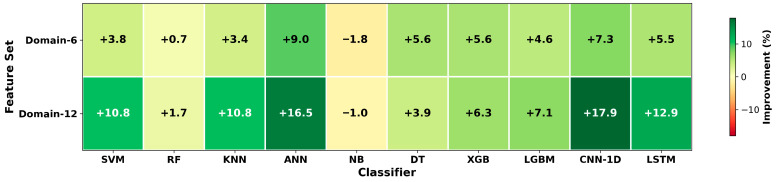
Improvement rates (%) relative to Baseline.

Figure 3 shows the percentage improvements in the Domain-6 and Domain-12 feature sets relative to Baseline. Positive values (green) indicate performance gains, while negative values (red) indicate performance drops. In the figures, XGB denotes XGBoost.

**Figure 4:**
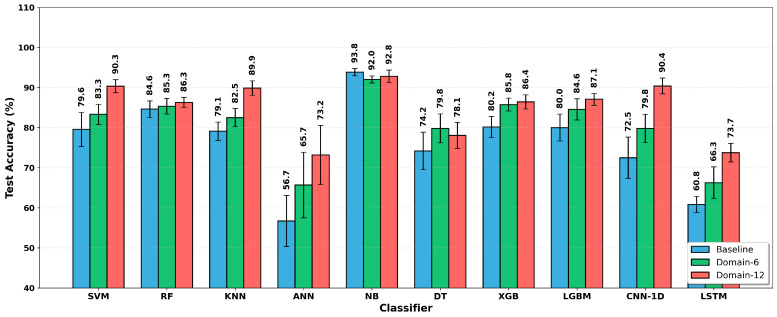
Feature engineering method comparison: test accuracy and standard deviation.

Figure 4 provides a comparative bar chart of the three feature sets across all classifiers. The error bars represent the standard deviation over 30 independent runs.

In Figure 2, the consistently dark-green appearance of the Naive Bayes row across all columns indicates strong generalization performance, largely independent of the feature set (approximately 92–94%). The red tones observed for ANN and LSTM reflect learning difficulties on small datasets. As shown in Figure 3, substantial improvements are observed for CNN-1D, ANN, LSTM, SVM, and KNN. It is also clear that these gains increase markedly as the number of statistical features grows. Figure 4 further shows that, except for NB and DT, most algorithms exhibit some improvement with Domain-6 and considerably larger improvements with Domain-12. This effect elevates models such as SVM and KNN to higher performance levels and enables CNN-1D to move from relatively low performance to outperform several conventional algorithms.

**Figure 5:**
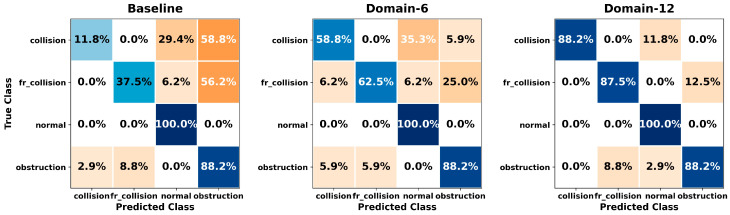
Confusion matrices for the SVM classifier.

**Figure 6:**
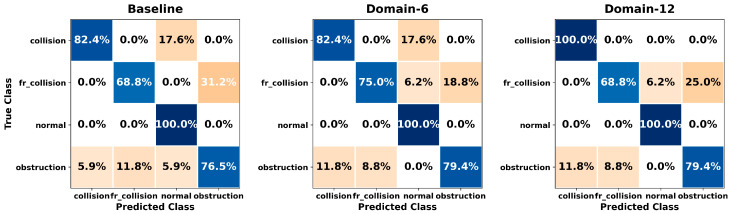
Confusion matrices for the RF classifier.

**Figure 7:**
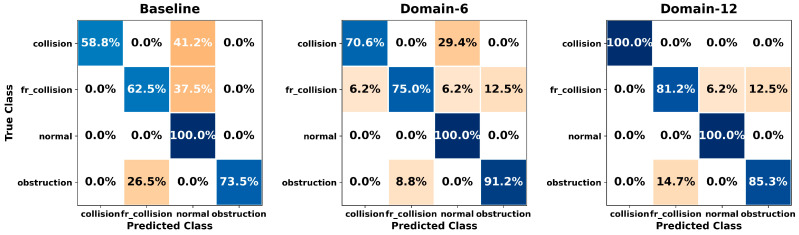
Confusion matrices for the KNN classifier.

**Figure 8:**
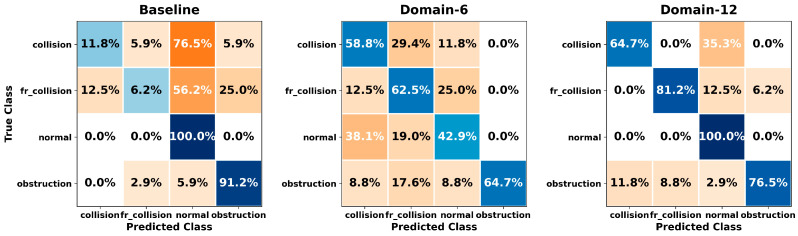
Confusion matrices for the ANN classifier.

**Figure 9:**
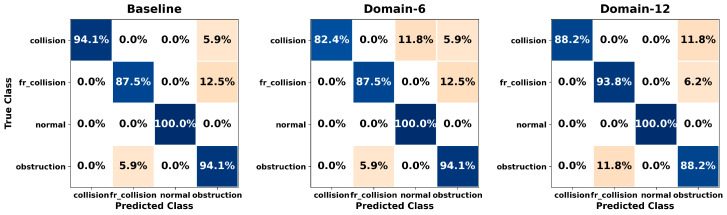
Confusion matrices for the NB classifier.

**Figure 10:**
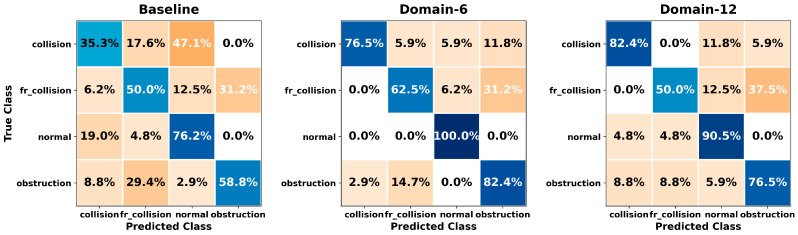
Confusion matrices for the DT classifier.

**Figure 11:**
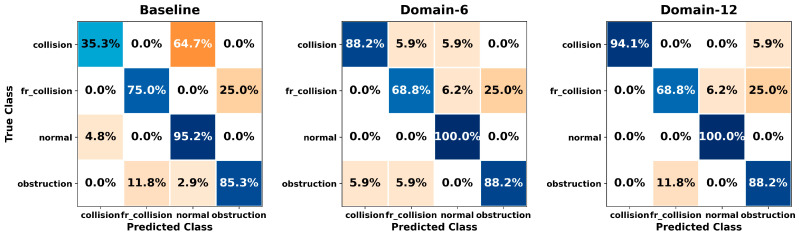
Confusion matrices for the XGB classifier.

**Figure 12:**
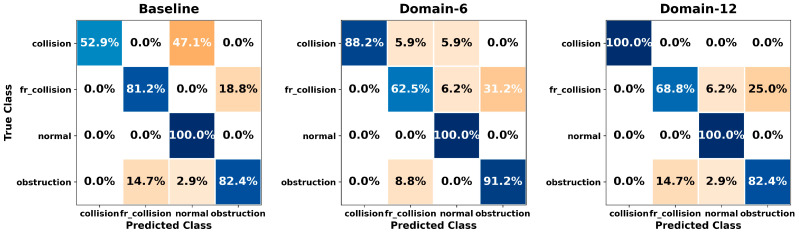
Confusion matrices for the LGBM classifier.

**Figure 13:**
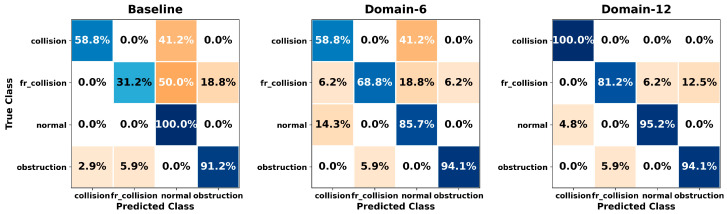
Confusion matrices for the CNN-1D classifier.

**Figure 14:**
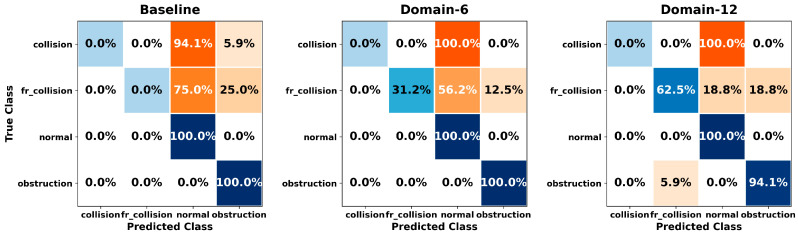
Confusion matrices for the LSTM classifier.

Figure 5, Figure 6, Figure 7, Figure 8, Figure 9, Figure 10, Figure 11, Figure 12, Figure 13 and Figure 14 present the confusion matrices of the SVM, RF, KNN, ANN, NB, DT, XGB, LGBM, CNN-1D, and LSTM classifiers, respectively, for the Baseline, Domain-6, and Domain-12 feature sets. Blue cells indicate correct classifications, while orange cells indicate misclassifications. Darker shading corresponds to higher percentages of correct or incorrect predictions.

The confusion matrices in Figure 5, Figure 6, Figure 7, Figure 8, Figure 9, Figure 10, Figure 11, Figure 12, Figure 13 and Figure 14 provide a detailed, class-wise view of each classifier’s prediction performance across the three feature sets. Diagonal entries represent correct classifications, whereas off-diagonal entries indicate confusion between classes. In general, Normal and Obstruction classes achieve the highest recognition rates across most algorithms, likely due to their larger sample sizes. In contrast, frequent confusion is observed between Front Collision and Collision, suggesting that these two failure types produce similar force–torque signatures. Notably, this confusion is reduced with Domain-6 and decreases substantially with Domain-12, accompanied by higher prediction rates. The overall increase in diagonal values and the decrease in off-diagonal values for Domain-12 confirm that advanced statistical features strengthen inter-class separability. Moreover, the more balanced diagonal distribution achieved by CNN-1D compared to LSTM indicates that the convolutional architecture captures local patterns in the force–torque signals more effectively.

### 3.2. Permutation Feature Importance Analysis

Permutation feature importance is a model-agnostic interpretability technique that assesses how strongly a model’s predictive performance depends on a given feature. In this approach, the values of a feature are randomly shuffled, breaking the original relationship between the feature and the target, and the resulting performance drop is measured. The larger the drop, the more critical the feature is considered. In practice, the model is first trained and a reference accuracy is computed on the test set. Then, the values of the selected feature in the test set are randomly permuted and the model is re-evaluated on this corrupted data. The difference between the reference and post-permutation accuracies constitutes the feature importance score. This process is repeated for all features to obtain a comprehensive ranking of importance.

**Figure 15:**
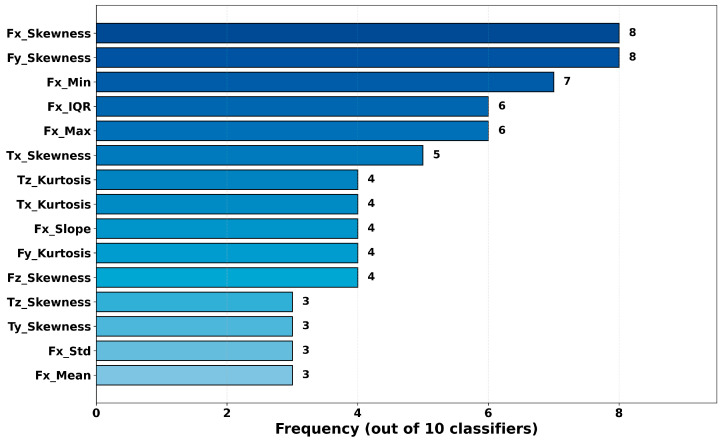
Most frequent features across all classifiers.

**Figure 16:**
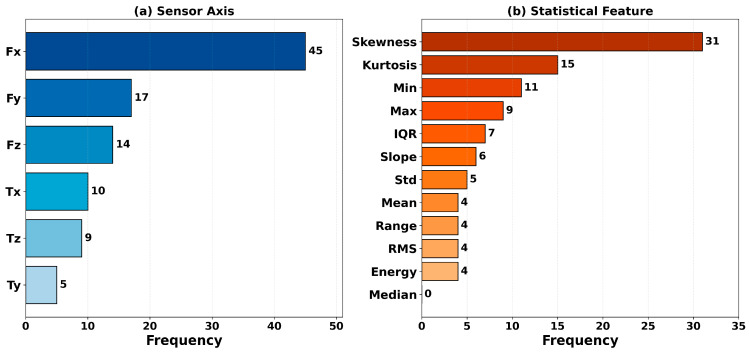
Feature frequency by sensor axis and statistical feature type.

**Figure 17:**
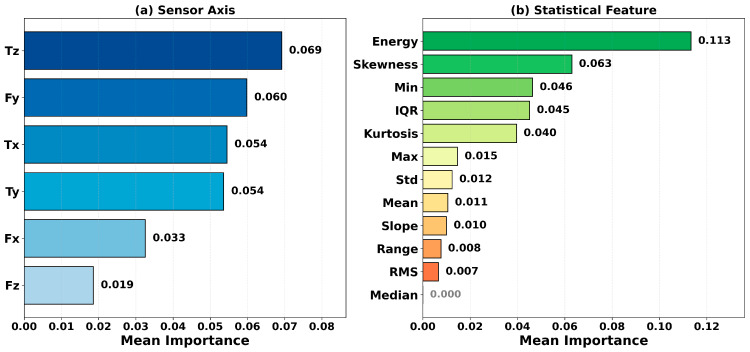
Average feature importance by sensor axis and statistical feature type.

In this study, permutation importance is computed for all classifiers using the Domain-12 feature set. For each classifier, the top 10 features with the highest importance scores are identified, and the results are analyzed from two perspectives: frequency analysis and mean-importance analysis. Frequency analysis evaluates the general relevance of a feature by counting how many classifiers include it in their top-10 list. In contrast, mean-importance analysis measures absolute impact by averaging the accuracy decrease attributable to that feature across classifiers. Considering these two measures jointly enables identification of features that are both consistently important and associated with large effect sizes. The most frequently occurring features across all classifiers are shown in Figure 15, the frequency distribution by sensor axis and statistical feature type is shown in Figure 16, and the average feature importance values are presented in Figure 17.

To clarify the terminology: “frequency by sensor axis” refers to how many times features derived from a particular sensor channel (Fx, Fy, Fz, Tx, Ty, Tz) appear in the top-10 importance lists across all classifiers. For example, as shown in Figure 16, features derived from the Fx axis exhibit the highest frequency among all sensor axes, indicating that this sensor axis carries critical information for failure detection. Similarly, “frequency by statistical type” indicates how many times a particular statistical descriptor (mean, std, skewness, kurtosis, etc.) appears in the top-10 lists across all classifiers.

As shown in Figure 15, Fx_Skewness and Fy_Skewness appear in the top-10 lists of 8 out of 10 classifiers, exhibiting the highest frequency. This finding indicates that skewness plays a critical role in characterizing failure conditions, suggesting that the asymmetry of the force distribution is an important discriminative cue between normal operation and failures. Figure 16 shows that the dominance of Fx and Fy among sensor axes implies that horizontal-plane forces are more informative for failure detection than vertical forces and torques. Among statistical features, the prominence of skewness and kurtosis highlights the discriminative power of distribution-shape descriptors. Figure 17 provides a complementary perspective by reporting mean importance scores rather than frequencies. Notably, the energy feature exhibits the highest average importance (0.113). Although it does not rank among the most frequent features, it has a significant impact in the models that include it. This points to the presence of “sparse but strong” features and underscores the value of comprehensive feature evaluation. As a notable finding, only 15 out of 72 features (21%) in the Domain-12 feature set appear in the top-10 list of more than two classifiers. This result indicates that not all statistical features are equally discriminative, and distribution shape descriptors such as skewness and kurtosis are particularly critical for robot failure detection. This finding supports that the domain-based feature engineering approach, unlike random feature generation, produces physically meaningful and discriminative features.

Also, the dominance of skewness features (Fx_Skewness, Fy_Skewness) over energy-based features (energy, RMS) deserves physical interpretation. Skewness measures force distribution asymmetry, which encodes the temporal structure of robot–environment interactions. During normal operation, force profiles tend to be symmetric as the robot executes smooth movements. Failure conditions–collisions, obstructions, front collisions—introduce characteristic asymmetries: sharp unidirectional force spikes followed by gradual decay (positive skewness) or sustained buildup followed by abrupt release (negative skewness). In contrast, energy and RMS measure signal magnitude, which can be similar across different states (e.g., moving heavy payloads vs. collision forces). Thus, skewness captures the character of interactions rather than just their magnitude, explaining its superior discriminative power.

## 4. Conclusions and Discussion

In this study, a comparative classification framework is conducted to identify robot failures using data collected from force–torque sensors. The results clearly demonstrate that feature engineering has a critical impact on classification performance. The consistent improvements achieved by the Domain-12 feature set across many algorithms indicate that statistical features inspired by modern signal-processing literature provide more discriminative representations than raw time-series inputs. In particular, incorporating advanced statistics such as skewness, kurtosis, and energy captures the characteristic signatures of failure conditions more effectively.

The source of performance improvement observed between Baseline (90 raw features) and Domain-12 (72 statistical features) requires careful analysis. This improvement cannot be explained solely by dimensionality reduction, as random selection of 72 features would not produce similar results. The fundamental sources of improvement are: (1) Domain knowledge: Skewness reflects the asymmetry of force distribution, kurtosis captures sudden changes, and energy represents signal power—these features capture the physical characteristics of failure conditions. (2) Noise filtering: Statistical features naturally filter measurement noise by being computed over 15 time steps. (3) Discriminative power: Permutation importance analysis reveals that only 15 out of 72 features (21%) show consistent importance, and these features (skewness, energy) are advanced statistics specific to Domain-12. These findings demonstrate that the proposed feature engineering approach, unlike random feature selection, produces domain-informed features that carry critical information for failure detection.

When algorithmic performance is considered, the consistently strong results of the Naive Bayes classifier across all feature sets are noteworthy. The strong NB performance despite force–torque sensors being inherently correlated warrants explanation. NB can succeed even when independence is violated because optimal classification requires only correct ranking of posterior probabilities, not accurate probability estimates. Moreover, domain features (Fx_Skewness, Tz_Median, etc.) exhibit lower correlation than consecutive raw time steps, partially addressing the violation. The finding that only 21% of features are consistently important suggests NB relies on a relatively independent feature subset. Gradient-boosting methods (XGBoost and LightGBM) also achieve substantial performance, especially with richer feature sets, corroborating the effectiveness of ensemble learning in modeling complex decision boundaries. Among deep learning models, the clear superiority of CNN-1D over LSTM indicates that extracting local patterns from force–torque signals is more beneficial than modeling long-term dependencies in this setting. Moreover, SVM and KNN achieved around 90% classification accuracy with the Domain-12 feature set, ranking among the top four methods alongside NB and CNN-1D.

The potential impact of class imbalance on classification performance should also be considered. In the dataset, the Collision and Front Collision classes are smaller in size compared to the Obstruction class. This may increase the tendency of smaller classes to be misclassified into larger classes, particularly in classifiers that rely on prior probabilities such as Naive Bayes. The confusion matrices in Figure 9 partially reflect this pattern. Nevertheless, the main finding of the study—the consistent improvement provided by the Domain-12 feature set—is observed across all classifiers and all classes. This indicates that the proposed feature engineering approach is effective regardless of class distribution. In future work, a more detailed examination of this effect using class balancing techniques (SMOTE, class weighting) is planned.

When comparing with previous studies in the literature, methodological differences should be considered. In the original study [1] that introduced the LP1 dataset, feature transformations such as windowed averages and derivatives were used with SKIL and OC1 algorithms. However, since different feature representations, different algorithms, and different evaluation protocols were used, direct accuracy comparison is not methodologically appropriate. The value of this study lies not in absolute accuracy values but in the controlled experimental design: Under the same dataset and same evaluation protocol, improvements achieved by merely changing the feature representation reveal the isolated effect of feature engineering. This approach demonstrates the effectiveness of domain-based statistical features in robot failure detection, independent of accuracy competition with previous studies.

Permutation importance analysis reveals that skewness features derived from the Fx and Fy channels are the most critical variables for failure detection. The dominant contribution of horizontal-plane forces suggests that anomalies along these axes best reflect failure-related interactions between the robot arm and its environment. In addition, the observation that the energy feature has the highest average importance, despite not ranking among the top in frequency, highlights the need for comprehensive feature evaluation. Overall, this study demonstrates that domain-based statistical feature engineering offers an effective approach for robot failure classification and that substantial improvements can be achieved in both conventional machine learning and deep learning models when appropriate feature representations are employed.

The study also has some limitations. The relatively small size of the dataset used (N = 88, 4 classes) restricts deep learning models from fully realizing their potential. The generalizability of results obtained on small datasets is limited, and validation studies on different datasets are required. Nevertheless, this limitation does not affect the main contribution of the study: On the same limited dataset, improvements are achieved by merely changing the feature representation. These findings demonstrate that feature engineering is critically important even under limited data conditions. Since collecting labeled failure data in industrial robotics applications is typically costly and time-consuming, the ability to achieve high performance with small datasets is practically valuable.

Finally, future work should therefore consider evaluations on larger and more diverse datasets, further investigate transfer learning strategies, and validate real-time deployment feasibility. Regarding computational efficiency, Domain-12 requires additional computation for sorting (median, IQR) and higher-order moments compared to Domain-6, but remains computationally lightweight for typical embedded systems. For real-time applications, feature subset selection—using only the 15 most important features identified by permutation importance—can significantly reduce latency while retaining most discriminative power. Hardware acceleration (FPGA/GPU) offers further optimization opportunities for ultra-low-latency scenarios.

## Figures and Tables

**Table 1:** Main characteristics of the dataset.

Feature	Value/Description
Dataset name	Approach to Grasp Position
Total number of samples	88
Number of raw features	90 (6 sensors × 15 time steps)
Number of classes	4 (normal, collision, front collision, obstruction)
Sensor channels	Fx, Fy, Fz (force); Tx, Ty, Tz (torque)
Time horizon	15 consecutive measurements

**Table 2:** Class distribution in the dataset.

Code	Class Name	Response Name	Sample Size	Class Distribution
0	Normal	normal	21	23.86%
1	Collision	collision	17	19.32%
2	Front Collision	fr_collision	16	18.18%
3	Obstruction	obstruction	34	38.64%
		Total	88	100%

**Table 3:** Feature sets and their dimensionalities.

Feature Set	Number of Features	Description
Baseline	90	6 sensors × 15 time steps (raw data)
Domain-6	36	6 sensors × 6 statistics (basic statistics)
Domain-12	72	6 sensors × 12 statistics (extended statistics)

**Table 4:** Domain features, descriptions, and associated feature sets.

No.	Feature	Description	Set
1	Mean	Arithmetic mean: central tendency of the signal	Domain-6, 12
2	Std	Standard deviation: signal variability	Domain-6, 12
3	Min	Minimum value: lower bound of the signal	Domain-6, 12
4	Max	Maximum value: upper bound of the signal	Domain-6, 12
5	Range	Value range: signal amplitude	Domain-6, 12
6	Slope	Trend/slope of the time series	Domain-6, 12
7	Median	Middle value of sorted data; robust to outliers	Domain-12
8	Skewness	Measure of distribution asymmetry	Domain-12
9	Kurtosis	Distribution tail heaviness/peakedness	Domain-12
10	RMS	Root mean square; signal magnitude	Domain-12
11	Energy	Total energy; signal power content	Domain-12
12	IQR	Interquartile range; robust spread	Domain-12

**Table 5:** Cross-validation and training parameters.

Parameter/Method	ML (8 Models)	DL (2 Models)
Evaluation method	5 × 5 Nested CV	5-Fold CV + Validation Split (20%)
Number of outer folds	5	5
Number of inner folds	5	None (fixed architecture)
Hyperparameter tuning	RandomizedSearchCV (20 iterations)	None (fixed architecture)
Regularization	Via hyperparameters	Early stopping (patience = 5)
Optimizer	Algorithm-specific	Adam
Loss function	Algorithm-specific	Categorical cross-entropy
Selection criterion	Inner-CV accuracy	Validation loss
Number of independent runs	30	30
Total evaluations (per classifier)	30 runs × 5 outer folds = 150	30 runs × 5 outer folds = 150
Total model training	150 × (20 iters × 5 inner folds + 1) ≈ 15,150	150 × 1 = 150
Stratified sampling	Yes	Yes
Random seed initialization	Base seed = 42 (+1 increment per run)	Base seed = 42 (+1 increment per run)

**Table 6:** Hyperparameter search space and most frequently selected settings for machine learning algorithms.

Algorithm	Hyperparameter	Search Space	Most Frequent	Description
SVM	C	[0.1, 1, 10, 100, 1000]	1000	Regularization parameter
	kernel	[‘rbf’, ‘linear’, ‘poly’]	rbf	Kernel function type
	gamma	[‘scale’, ‘auto’, 0.001, 0.01, 0.1, 1]	0.1	Kernel coefficient (nonlinear only)
RF	n_estimators	[50, 100, 200, 300]	100	Number of trees in the forest
	max_depth	[None, 10, 20, 30]	30	Maximum tree depth
	min_samples_split	[2, 5, 10]	2	Minimum samples to split a node
	min_samples_leaf	[1, 2, 4]	1	Minimum samples per leaf
KNN	n_neighbors	[1, 3, 5, 7, 9, 11]	1	Number of neighbors (k)
	weights	[‘uniform’, ‘distance’]	distance	Neighbor weighting scheme
	metric	[‘euclidean’, ‘manhattan’]	euclidean	Distance metric
ANN	hidden_layer_sizes	[(50), (100), (50,50), (100,50)]	(100, 50)	Hidden layer/unit configuration
	activation	[‘relu’, ‘tanh’]	relu	Activation function
	alpha	[0.0001, 0.001, 0.01]	0.01	L2 regularization coefficient
	learning_rate	[‘constant’, ‘adaptive’]	constant	Learning-rate schedule
NB	var_smoothing	[10^−9^, 10^−8^, 10^−7^, 10^−6^, 10^−5^]	10^−9^	Variance smoothing parameter
DT	max_depth	[None, 5, 10, 15, 20]	20	Maximum tree depth
	min_samples_split	[2, 5, 10]	2	Minimum samples to split a node
	min_samples_leaf	[1, 2, 4]	1	Minimum samples per leaf
	criterion	[‘gini’, ‘entropy’]	entropy	Split-quality criterion
XGBoost	n_estimators	[50, 100, 200]	200	Number of boosting iterations
	max_depth	[3, 5, 7, 10]	7	Maximum tree depth
	learning_rate	[0.01, 0.05, 0.1, 0.2]	0.2	Learning rate (shrinkage)
	subsample	[0.6, 0.8, 1.0]	0.6	Subsampling ratio
LGBM	n_estimators	[50, 100, 200]	100	Number of boosting iterations
	max_depth	[3, 5, 7, 10, −1]	10	Maximum tree depth
	learning_rate	[0.01, 0.05, 0.1, 0.2]	0.2	Learning rate
	num_leaves	[15, 31, 63]	31	Number of leaves

**Table 7:** Deep learning model architectures.

Model	Layers in Sequence	Parameters
CNN-1D	Conv1D	32 filters, kernel = 3, ReLU
	BatchNormalization	—
	MaxPooling1D	pool_size = 2
	Dropout	rate = 0.3
	Conv1D	64 filters, kernel = 3, ReLU
	BatchNormalization	—
	Dropout	rate = 0.3
	Flatten	—
	Dense	32 units, ReLU
	Dropout	rate = 0.4
	Dense (Output)	4 units, Softmax
LSTM	LSTM	32 units
	Dropout	rate = 0.3
	Dense	16 units, ReLU
	Dropout	rate = 0.4
	Dense (Output)	4 units, Softmax

**Table 8:** Performance metrics and their computation.

Metric	Computation
Accuracy	(TP + TN)/(TP + TN + FP + FN)
Precision	TP/(TP + FP)
Recall	TP/(TP + FN)
F1-Score	2 × (Precision × Recall)/(Precision + Recall)

**Table 9:** Performance results of the classification algorithms using the Baseline feature set.

Classifier	Accuracy (%)	Precision (%)	Recall (%)	F1-Score (%)	Total Time (Min)
SVM	79.57 ± 4.25 [67.06, 86.41]	78.58 ± 7.38 [57.57, 88.21]	74.87 ± 5.06 [60.06, 83.87]	73.00 ± 6.28 [54.66, 83.51]	3.79
RF	84.63 ± 2.05 [80.65, 88.50]	86.39 ± 2.55 [81.19, 89.82]	84.09 ± 2.03 [79.23, 86.85]	83.16 ± 2.26 [77.93, 86.80]	91.39
KNN	79.10 ± 2.30 [74.90, 83.01]	82.38 ± 3.09 [72.59, 86.42]	79.00 ± 2.40 [72.92, 83.21]	76.79 ± 2.96 [68.75, 81.56]	8.01
ANN	56.72 ± 6.40 [43.59, 68.10]	42.98 ± 7.29 [28.59, 58.33]	49.87 ± 5.22 [40.48, 60.89]	42.96 ± 6.27 [29.73, 54.97]	8.15
NB	93.85 ± 0.90 [92.03, 95.42]	95.43 ± 0.74 [94.18, 96.92]	93.45 ± 0.89 [91.79, 95.12]	93.51 ± 0.97 [91.85, 95.48]	1.88
DT	74.19 ± 4.67 [56.93, 80.52]	75.71 ± 5.29 [52.51, 82.04]	73.42 ± 4.36 [55.01, 79.29]	71.54 ± 4.73 [52.28, 77.37]	2.52
XGB	80.16 ± 2.61 [75.10, 85.42]	81.48 ± 4.68 [69.52, 89.05]	77.29 ± 3.31 [70.95, 84.11]	75.87 ± 3.87 [67.57, 82.89]	42.68
LGBM	80.02 ± 3.35 [73.73, 86.41]	81.48 ± 3.99 [71.75, 89.96]	78.40 ± 3.67 [71.86, 85.36]	76.78 ± 4.14 [69.67, 84.77]	15.18
CNN-1D	72.50 ± 5.16 [56.80, 80.98]	70.91 ± 7.85 [50.71, 84.39]	68.94 ± 5.77 [53.45, 78.93]	65.29 ± 6.98 [46.30, 77.85]	167.11
LSTM	60.81 ± 2.03 [55.75, 63.73]	34.03 ± 2.64 [29.50, 39.50]	49.44 ± 1.54 [45.48, 51.67]	38.39 ± 1.58 [34.51, 41.25]	169.08

**Table 10:** Performance results of the classification algorithms using the Domain-6 feature set.

Classifier	Accuracy (%)	Precision (%)	Recall (%)	F1-Score (%)	Total Time (Min)
SVM	83.33 ± 2.50 [78.43, 88.76]	85.38 ± 3.68 [75.84, 90.47]	80.75 ± 2.48 [74.29, 86.90]	80.01 ± 3.12 [70.75, 86.55]	1.88
RF	85.34 ± 1.96 [80.72, 89.67]	86.08 ± 2.10 [80.54, 89.54]	85.75 ± 1.96 [81.67, 89.35]	84.31 ± 2.26 [79.34, 88.60]	71.58
KNN	82.49 ± 2.26 [77.39, 86.34]	84.32 ± 2.72 [77.75, 89.70]	80.95 ± 2.17 [76.25, 85.36]	79.81 ± 2.35 [74.39, 84.42]	4.17
ANN	65.68 ± 8.20 [49.02, 87.58]	59.94 ± 13.35 [27.32, 87.76]	61.51 ± 9.35 [41.19, 84.70]	56.56 ± 11.55 [30.86, 84.47]	7.37
NB	92.05 ± 0.87 [90.78, 94.38]	93.68 ± 1.30 [88.66, 95.95]	91.43 ± 1.04 [89.52, 93.69]	91.30 ± 1.12 [89.15, 93.68]	1.08
DT	79.83 ± 3.59 [72.68, 86.21]	81.39 ± 3.25 [75.40, 87.47]	79.29 ± 3.09 [72.92, 85.19]	78.18 ± 3.28 [71.63, 83.58]	1.61
XGB	85.77 ± 1.60 [82.75, 88.69]	86.90 ± 2.25 [81.12, 90.72]	84.69 ± 1.82 [80.48, 88.27]	84.02 ± 1.78 [79.41, 87.49]	30.43
LGBM	84.58 ± 2.63 [80.39, 88.63]	85.96 ± 2.65 [80.10, 90.68]	83.74 ± 2.53 [78.76, 88.51]	83.06 ± 2.81 [77.82, 88.12]	10.55
CNN-1D	79.80 ± 3.53 [71.70, 86.27]	79.62 ± 5.59 [62.12, 90.07]	76.44 ± 4.07 [67.32, 83.15]	74.00 ± 4.97 [61.08, 82.83]	79.18
LSTM	66.27 ± 3.93 [60.13, 76.08]	49.13 ± 9.34 [38.18, 76.40]	56.81 ± 5.11 [49.52, 69.29]	48.83 ± 6.74 [39.78, 66.48]	78.00

**Table 11:** Performance results of the classification algorithms using the Domain-12 feature set.

Classifier	Accuracy (%)	Precision (%)	Recall (%)	F1-Score (%)	Total Time (Min)
SVM	90.33 ± 1.64 [86.47, 93.20]	91.69 ± 2.49 [84.62, 95.50]	90.26 ± 1.83 [85.60, 92.74]	89.80 ± 1.94 [84.16, 92.89]	4.77
RF	86.29 ± 1.29 [83.01, 88.50]	87.42 ± 1.34 [83.71, 90.14]	86.60 ± 1.19 [83.75, 88.93]	85.53 ± 1.29 [82.26, 87.95]	85.53
KNN	89.90 ± 1.80 [85.36, 93.27]	90.36 ± 2.05 [85.96, 94.48]	90.68 ± 1.96 [85.60, 94.64]	89.55 ± 2.08 [83.78, 93.36]	9.50
ANN	73.19 ± 7.34 [56.86, 87.39]	71.38 ± 10.23 [49.83, 89.91]	71.07 ± 7.92 [56.13, 85.77]	67.31 ± 9.75 [47.44, 85.99]	10.66
NB	92.83 ± 1.55 [88.82, 95.49]	94.25 ± 1.36 [91.04, 96.75]	93.16 ± 1.66 [89.11, 95.65]	92.61 ± 1.77 [88.51, 95.37]	3.59
DT	78.05 ± 3.25 [70.52, 84.18]	79.14 ± 3.17 [71.52, 85.12]	78.08 ± 3.16 [67.26, 82.63]	76.57 ± 3.33 [65.69, 81.80]	4.23
XGB	86.43 ± 1.76 [81.90, 89.80]	88.14 ± 1.86 [83.64, 91.84]	85.50 ± 1.72 [81.01, 88.27]	85.11 ± 2.03 [80.52, 88.35]	38.16
LGBM	87.07 ± 1.48 [82.94, 89.80]	87.88 ± 1.47 [85.39, 90.71]	86.77 ± 1.75 [82.92, 89.35]	86.12 ± 1.75 [82.27, 89.06]	14.77
CNN-1D	90.40 ± 1.97 [85.36, 94.38]	91.30 ± 2.67 [83.62, 95.88]	89.49 ± 2.50 [83.69, 93.33]	89.00 ± 2.74 [82.30, 94.03]	147.39
LSTM	73.75 ± 2.33 [68.17, 78.37]	63.48 ± 5.79 [53.88, 75.55]	67.74 ± 2.76 [61.73, 75.48]	61.92 ± 3.68 [55.95, 71.48]	146.29

## Data Availability

The dataset used in this study is publicly available; see Ref. [16] for details on data access.

## Abbreviations

The following abbreviations are used in this manuscript:

RMS	Root Mean Square
IQR	Interquartile Range
SVM	Support Vector Machines
RF	Random Forest
KNN	k-Nearest Neighbors
ANN	Artificial Neural Network
NB	Naive Bayes
DT	Decision Tree
XGBoost	eXtreme Gradient Boosting
LightGBM	Light Gradient Boosting Machine
LGBM	Light Gradient Boosting Machine
CNN-1D	One-Dimensional Convolutional Neural Network
LSTM	Long Short-Term Memory
SKIL	Structured Knowledge by Inductive Learning
OC1	Oblique Classifier 1
IoRT	Internet of Robotic Things
RNN	Recurrent Neural Network
TL	Transfer Learning
DCNN	Deep Convolutional Neural Network
DA	Data Augmentation
MCSA	Motor Current Signature Analysis
GRU	Gated Recurrent Unit
RV	Rotate Vector
Std	Standard Deviation
LP1	Learning Problem 1
RBF	Radial Basis Function
MLP	Multi-Layer Perceptron
ReLU	Rectified Linear Unit
CART	Classification and Regression Trees
NCV	Nested Cross-Validation
CV	Cross-Validation
